# A Review of Traditional and Emerging Residual Chlorine Quenchers on Disinfection By-Products: Impact and Mechanisms

**DOI:** 10.3390/toxics11050410

**Published:** 2023-04-26

**Authors:** Xue Li, Zhijing Zhao, Zheng Qu, Xinyu Li, Zengli Zhang, Xiaojun Liang, Jingsi Chen, Jiafu Li

**Affiliations:** 1 School of Public Health, Suzhou Medical College, Soochow University, Suzhou 215000, China; 2 Kunshan Center for Disease Control and Prevention, Suzhou 215301, China

**Keywords:** drinking water, disinfection by-products, residual chlorine, chlorine quenchers

## Abstract

Disinfection by-products (DBPs) are the most common organic contaminants in tap water and are of wide concern because of their highly developmental toxic, cytotoxic, and carcinogenic properties. Typically, to control the proliferation of pathogenic microorganisms, a certain concentration of residual chlorine is retained in the factory water, which reacts with the natural organic matter and the disinfection by-products that have been formed, thus affecting the determination of DBPs. Therefore, to obtain an accurate concentration, residual chlorine in tap water needs to be quenched prior to treatment. Currently, the most commonly used quenching agents are ascorbic acid, sodium thiosulfate, ammonium chloride, sodium sulfite, and sodium arsenite, but these quenching agents can cause varying degrees of DBPs degradation. Therefore, in recent years, researchers have attempted to find emerging chlorine quenchers. However, no studies have been conducted to systematically review the effects of traditional quenchers and new ones on DBPs, as well as their advantages, disadvantages, and scope of application. For inorganic DBPs (bromate, chlorate, and chlorite), sodium sulfite has been proven to be the ideal chlorine quencher. For organic DBPs, although ascorbic acid caused the degradation of some DBPs, it remains the ideal quenching agent for most known DBPs. Among the studied emerging chlorine quenchers, n-acetylcysteine (NAC), glutathione (GSH), and 1,3,5-trimethoxybenzene are promising for their application as the ideal chlorine quencher of organic DBPs. The dehalogenation of trichloronitromethane, trichloroacetonitrile, trichloroacetamide, and bromochlorophenol by sodium sulfite is caused by nucleophilic substitution reaction. This paper takes the understanding of DBPs and traditional and emerging chlorine quenchers as a starting point to comprehensively summarize their effects on different types of DBPs, and to provide assistance in understanding and selecting the most suitable residual chlorine quenchers during DBPs research.

## 1. Introduction

Water plays an important role in our lives, and its quality significantly impacts public health. In order to kill harmful microorganisms in water and prevent the spread of diseases from water, the disinfection of drinking water becomes a necessary process. However, in this process, disinfectants, natural organic matter in water, and inorganic ions (bromine, iodine ions) will react to produce disinfection by-products (DBPs) [1,2]. Recent toxicological studies have shown that DBPs are usually cytotoxic, genotoxic, and carcinogenic [1,2]. In addition, DBPs have higher biological toxicity and detectable concentrations compared to artificial contaminants in drinking water [3]. Therefore, it is essential to control DBPs in drinking water to reduce their risk to human health, which is conducive to the safety of drinking water and to improve the quality of drinking water.

Robust and sensitive methods for DBP analysis and proper sample handling procedures are essential to obtain accurate and reliable data on DBP occurrence, formation, and transformation. In most cases, drinking water is not directly analyzed after collection and needs to be stored for some time. Therefore, to prevent the continued generation of DBPs or the deconstruction of DBPs caused by residual chlorine, it should be quenched before storage [4]. Therefore, using appropriate quenching agents is critical to maintaining the stability of DBPs during the period between sample collection and analysis. Currently, sodium sulfite, sodium arsenite, sodium borohydride, ascorbic acid, and ammonium chloride are widely used as quenching agents [4]. These quenchers are low-cost, simple, and stable, and are widely recognized as common quenching agents with good quenching effects for special DBPs; however, they also have disadvantages, such as incomplete quenching (ammonium chloride) and a narrow quenching range [5]. The new quenchers, such as NAC, GSH, and silver-hydrogen peroxide, have also been found to have better quenching effects and quenching approaches [6]. They have certain levels of innovation and development compared with traditional quenching agents, and can essentially achieve the goals of cost reduction, complete quenching, and wide-range quenching, which provide feasible options for the effective control of DBPs formation.

However, to date, no study has attempted to provide a comprehensive summary of quenching agents, which is an important issue that has recently emerged. This paper reviews the effects of traditional and emerging chlorine quenchers on the degradation and toxicity of DBPs, which will help researchers to explore new quenchers and to select suitable chlorine quenchers during formation, transformation, and occurrence studies of DBPs.

## 2. Effect of Traditional Quenching Agents on Disinfection By-Products

The advantages and disadvantages of conventional quenchers were briefly summarized in the previous section. In order to fully understand the traditional quenching agents, we developed the following review of common quenching agents (Table 1).

### 2.1. Ascorbic Acid

Recent relevant studies have suggested the use of ascorbic acid to quench organic DBPs, namely trihalomethanes (THMs), haloacetic acids (HAAs), haloacetonitriles (HANs), halogenated ketones (HKs), and halogenated acetaldehyde (HALs), which is suitable for most of the organic DBPs [4,5,7]. In addition, ascorbic acid does not affect the concentration of any polar iodinated DBPs, and ascorbic acid also reduces the cytotoxicity formed [8,9]. Ascorbic acid treatment can rapidly consume a large number of active halogen compounds, only producing inorganic halides and dehydroascorbic acid without additional halogenated organic molecules; therefore, ascorbic acid is a more ideal residual chlorine quencher when the analyte is organic DBPs.

Ascorbic acid can rapidly consume large amounts of residual oxidants through redox reactions (e.g., sodium hypochlorite within 1 min), producing much weaker inorganic halide by-products [4,10]. Recent studies have shown that ascorbic acid is stable to most the organic DBPs, including THMs, HAAs, HKs, HALs, and most of HANs, haloacetamides (HAMs), and phenolic DBPs [4,5,10]. However, ascorbic acid is not suitable as a quencher for inorganic DBPs, halofuran (MX), trichloroacetonitrile (TCAN), and trichloronitromethane (TCNM), especially chlorate [5].

In addition, Urbansky et al. (1999) found that chloral hydrate was significantly decomposed when ascorbic acid was used as a quenching agent before analysis [10]. However, the results of the recent studies on ascorbic acid on tricromoacetaldehyde (TCAL) are inconsistent, and studies from 2014, 2020, and 2021 showed that ascorbic acid did not affect chloral hydrate under acidic or alkaline conditions [4,7,11]. In addition, Moore et al. showed that, under neutral conditions, ascorbic acid caused a 10–30% increase in tribromoacetaldehyde (TBAL) [5].

Gao et al. (2020) found that TCAN and TCNM are two more specific DBPs because 65% and 37% of them will be degraded by ascorbic acid within 48 h under alkaline or neutral conditions (pH 7 or 8), respectively; but, under acidic conditions, there is no significant effect (pH < 5) [7]. In addition, Moore et al. (2021) showed that under neutral conditions, ascorbic acid had no effect on TCNM concentrations, but caused a seven-fold increase in bromodichloronitromethane (BDCNM) and dibromochloronitromethane (DBCNM) concentrations [5]. Furthermore, a 2021 study showed that, under neutral conditions, ascorbic acid caused degradation of MX and dibromoacetamide (DBAM), which reduced MX concentrations by 20% and 21% within 24 and 168 h, respectively, and also reduced DBAM concentrations by 8.9–16.4% in the same time [5].

Kristiana et al. (2014) showed through relevant experiments that the presence of ascorbic acid caused a significant decrease in the concentration of chlorite at pH 7 and 8. In particular, at pH 7, the concentration of chlorite decreased below the detection limit at t = 4 and 168 h [4]. The sharp decrease in chlorite concentration may be due to the interference of ascorbic acid in ion chromatography analysis, or the redox reaction between ascorbic acid and chlorite. Therefore, ascorbic acid should not be used as a quencher for chlorite analysis samples. During the experiments, the presence of ascorbic acid did not cause significant changes in the concentrations of HANs, except for TCAN and HKs, so ascorbic acid was suitable for the analysis of HKs and most HANs.

For aromatic DBPs, previous researchers have experimentally found that ascorbic acid did not affect the concentrations of halophenols, halohydroxybenzaldehydes, and halosalicylic acids [9,11]. However, ascorbic acid caused a 20% degradation of MX. Therefore, ascorbic acid is an ideal chlorine quencher for the analysis of aromatic DBPs, except for MX [9].

In general, ascorbic acid is a ideal quenching agent in most of the organic DBPs and does not cause their decomposition, but is not a suitable quenching agent for inorganic DBPs, TCNM, and MX.

### 2.2. Sodium Sulfite

Sodium sulfite is another common quenching agent and has an excellent quenching effect on residual chlorine. For inorganic DBPs and MX, sodium sulfite is an ideal quenching agent, but for many organic DBPs, their measurement can be adversely affected. In addition, sodium sulfite is effective against certain classes of drugs. Therefore, sodium sulfite can be used as a quenching agent for THMs, HAAs, inorganic and MX residual chlorine.

Sodium sulfite has no significant effect on any inorganic DBPs and can be used for simultaneous analysis of chlorite, chlorate, and bromate. Furthermore, sodium sulfite is the best quenching agent of MX. Meanwhile, sodium sulfite can be used to analyze HAAs and THMs. However, sodium sulfite also causes the degradation of unregulated priority and emerging DBPs, including HNMs, HANs, HKs, HALs, HAMs, haophenols, halopropanes, and halopropylenes [5]. This is because sulfite has a strong nucleophilic capacity, which reacts with DBPs via nucleophilic substitution reaction to form dechlorinated products [12,13,14,15].

For HNMs, the presence of sodium sulfite reduced 97% of TCNM (from 10 to 0.3 µg/L) at one hour at sample pH 7 and 8 [4], which is consistent with that reported by [13], who noted that in the presence of sodium sulfite, TCNM was reduced to dichloronitromethane (DCNM). The kinetics of the reaction are highly dependent on pH at a pseudo-first-order reaction.

For HALs, in the presence of sodium sulfite, at pH 7 and 8, the concentrations of TCAL, dichloroacetaldehyde (DCAL), and bromochloroacetaldehyde (BCAL) did not change much from 1 to 24 h; however, beyond 168 h, 40–60% of DCAL and BCAL were degraded. Furthermore, for bromodichloroacetaldehyde (BDCAL), dibromochloroacetaldehyde (DBCAL), and TBAL, they almost completely degraded after 168-h reaction [4].

For HKs, at pH 7, the presence of sodium sulfite reduced 96.5 1,3-dichloroacetone (1,3-DCP) during a 168-h reaction [4]. In addition, Munch et al. (1995) demonstrated that sodium sulfite promotes the decomposition of some HKs (1,1-dichloroacetone and 1,1,1-trichloroacetone) [14].

For HANs, sodium sulfite promotes their decomposition, such as the decomposition of TCAN to form dichloroacetonitrile (DCAN), and further hydrolyzes into dichloroacetic acid (DCAA). It was also found that the stability of dibromoacetamide (DBAN) was adversely affected by sodium sulfite [13,14,15]. Croue et al. (1989) reported that TCAN and DBAN can be rapidly destroyed in chlorinated drinking water by a small dose of sodium sulfite aqueous solution due to the dehalogenation of HANs caused by sodium sulfite [13].

For halopropanes, Munch et al. (1995) indicated that sodium sulfite promoted the decomposition of 1,1-dichloropropane, 1,1,1-trichloropropane, and propylene chloride, and recommended the use of ammonium chloride as an alternative quencher [14]. Bauman et al. (1989) showed that 3-bromopropene was significantly reduced by 89% compared to the initial reduction in the presence of sulfite [12].

For HAMs, when quenching well-chlorinated samples with sodium sulfite, HAMs in the samples were reduced to undetectable levels [5]. In addition, sodium sulfite also resulted in a decrease of total organic halogen (TOX) in the quenched sample. Stevens et al. (1985) noted a decrease in TOX concentration in stored surface water samples dechlorinated with sulfites and an increase in untreated samples [16]. The decay of the sample was attributed to the (possible) “decomposition of metastable organohalides formed during chlorination”. Stanbro et al. (1982) demonstrated that several organochlorines are not immediately reduced by sulfite as previously assumed, and may take several hours to dechlorinate [17]. For other aromatic DBPs, Li et al. (2021) proved that sodium sulfite caused dechlorination of chlorobromophenols [11]. Wu et al. (2012) found that sodium sulfite reduced the genotoxicity of chlorinated effluent from secondary wastewater treatment plants [18].

In general, sodium sulfite has an adverse effect on the stability of most organic DBPs among the quenchers tested and is more suitable as a quenching agent for inorganic DBPs and MX.

### 2.3. Ammonium Chloride

Unlike ascorbic acid and sodium sulfite, ammonium chloride does not react with free chlorine by redox reaction, but reacts with hypochlorous acid (HOCl) or hypochlorite anion (OCl^−^) to form chloramine, and its reaction with residual chlorine is described in Section 3.1 of this paper, and theoretically, no further DBPs are produced in this process. Ammonium chloride has less effect on TCNM than the previous two [4]. Ammonium chloride is the least destructive quencher for highly impacted N-DBPs and C-DBPs of sodium sulfite; for HAMs, HALs, HANs, etc., the effect of ammonium chloride on them depends on whether the sample is chlorinated or not; in addition, ammonium chloride has the advantage of controlling microbial growth in chlorinated samples, but ammonium chloride cannot reduce the cytotoxicity of chlorinated wastewater.

The EPA Method 551.1 and Standard Methods recommend the use of ammonium chloride to quench chlorine prior to the analysis of most organohalides [14]. Ammonium chloride is also used as a standard method for the analysis of HAAs (EPA Method 552.2) [14]. Ammonium chloride makes considerable errors in more DBPs during use, where chloroform is stored for 24 h and its concentration produces an error of 27–31% [5]. In addition, researchers have found that ammonium chloride causes the formation or degradation of several types of DBPs, such as HAAs [19], HNMs [20], and HAMs [21].

For THMs, quenched samples by ammonium chloride elevated the concentration of trichloromethane (TCM) by 27–31% compared to unquenched ones. For HANs and HNMs, the percentage errors were 24% and 72% for HANs and HNMs in unquenched samples after 7 days when chlorine was present, and 175% and 135% when chlorine was not present. DCAN, TCNM, and BCAN all increased when in the presence of ammonium chloride alone, and TCAN, BDCNM, and DBCNM decreased when ammonium chloride was used to quench chlorine [5]. In addition, for HNMs, compared to quenching agents such as sodium sulfite and ascorbic acid, ammonium chloride had the least effect on TCNM [4].

For HAMs and HALs, ammonium chloride does not statistically significantly change in the absence of chlorine, but when chlorine is present, the concentrations of TCAL and TBAL are 10–77% lower than unquenched, which may be caused by the instability of these compounds in the presence of residual chlorine [7,21]. Another study proved that only BCAL and BDCAL were adversely affected in the presence of ammonium chloride, and the concentrations of both decreased by 50% and 40%, respectively, during the experiment [4]. For dibromoacetamide (DBAM), there were relatively large errors for all quenching agents. Ascorbic acid marginally outperformed the others, but ammonium chloride may be effective at higher quenching agent doses [5].

### 2.4. The Impact of Traditional and Emerging Residual Chlorine Quenchers on Cytotoxicity

Through relevant experiments, Du et al. (2017) compared the effects of ascorbic acid, sodium thiosulfate (both reducing agents), and ammonium chloride (non-reducing agent) on the cytotoxicity of chlorinated wastewater, and found that the sodium thiosulfate and ascorbic acid reduced the cytotoxicity of chlorinated samples by 22–45% and 6–27%, respectively, while the non-reducing agent ammonium chloride did not reduce the cytotoxicity of chlorinated samples [8].

They speculated that it might be due to the reducing agent reducing the cytotoxicity of the unchlorinated wastewater; however, it was found by the experiment that ascorbic acid and sodium thiosulfate did not reduce the cytotoxicity of the unchlorinated wastewater; in addition, the residual chlorine did not cause the formation of cytotoxicity that would not be caused in ultrapure water during the solid phase extraction. This may be because the reducing quencher prevented the further formation of DBPs or prevented the reaction between DBPs and the quencher, thus reducing cytotoxicity [8,22].

## 3. Emerging Residual Chlorine Quenchers

With the study of quenchers, researchers are no longer satisfied with the commonly used quenchers such as sodium sulfite and ascorbic acid. They have started to look for new quenchers, which not only provide a new concept and direction for controlling the formation of DBPs, but also improve the quenching effect of the quenching agent and reduce the quenching cost in its research.

### 3.1. N-Acetylcysteine

In the search for new quenchers, reduced sulfur compounds (RSC), including n-acetylcysteine (NAC), and glutathione (GSH), were found to easily react with chlorine and chloramine. Based on the results of the present study, NAC is considered an ideal quencher for most DBP and TOX analyses, except for HNMs. The much higher reactivity of chlorine and chloramine to reducing sulfur groups in NAC protects other functional groups (e.g., alkyls, amines, and amides), thereby avoiding the formation of CX_3_R-DBPs and maintaining a stable concentration of DBPs [23,24]. Specifically, NAC applies as a quencher for THMs, HAAs, and DCAL, as well as 1,1,1-trichloropropanone (1,1,1-TCP) and TCAN, and can also be used to some extent for analyzing TCAL, DCAN, DBAN, dichloroacetamide (DCAM), and trichloroacetamide (TCAM), but NAC is not applicable to DCNM and TCNM analysis.

Ding et al. (2022) found that the concentration changes of THMs, HAAs, and HKs were negligible within 168 h (almost less than 10.0%) [6]. However, TCAL, DCAN, DBAN, DCAM, and TCAM have slight hydrolysis under the same conditions [25,26,27,28,29]. Although the presence of NAC (20.0 μmol/L) promotes the hydrolysis of TCAL, DCAN, DCAM, and TCAM, the influence of NAC can be ignored due to the comparable rate of reduction and dehalogenation, and the very low molar ratio of NAC to disinfectant in real factory water. Therefore, when NAC acts as a quencher for TCAL, DCAN, DBAN, DCAM, and TCAM before analysis, it is necessary to immediately analyze samples for TCAL, DCAN, DBAN, DCAM, and TCAM to minimize negative interference with hydrolysis. Concurrent experimental results showed an immediate hydrolysis of 1,1,1-TCP and TCAN, resulting in their rapid losses within a few hours at pH 7. However, the effect of NAC (20.0 μmol/L) on 1,1,1-TCP and TCAN stability is negligible. Thus, the addition of NAC as a quencher is suitable for the determination of 1,1,1-TCP and TCAN. Similarly, 1,1,1-TCP and TCAN should be measured as quickly as possible to avoid hydrolysis [6].

However, the destruction of DCNM and TCNM by NAC was obvious. In the absence of NAC, DCNM and TCNM were slightly hydrolyzed, but a significant reduction in DCNM and TCNM concentrations in the presence of NAC was observed. In NAC quenched samples, DCNM decomposed a 1.6-fold increase in k_obs_. TCNM had completely disappeared after 3 h in the presence of 20.0 μmol/L of NAC, which was also observed when TCNM was resolved by sodium sulfite and sodium thiosulfate [13]. The rapid degradation of DCNM and TCNM by NAC limits its application in the DCNM and TCNM analyses. Therefore, such samples should be immediately analyzed without adding any quencher.

In the experiments investigating the effects of various quenchers on TOX assays, a relatively low reduction in TOX was observed in samples quenched with NAC, and GSH for 3 h, ranging from 7.0% to 19.3%. With longer quenching time (24 h), TOX reduction in samples with NAC (8.0%) and glutathione (13.0–19.0%) were lower than those with sodium sulfite (30.0%) and sodium thiosulfate (36.0%) [6].

### 3.2. Glutathione (GSH)

GSH, which belongs to RSC, was also selected as a quencher. Both NAC and GSH have reduced sulfur groups, therefore, the effect of GSH on chloride and chloramine, organic DBPs, and TOX are the same as NAC.

When the molar ratio of Cl_2_ or NH_2_Cl to GSH is less than 0.5, the generation of CX_3_R-DBPs during the chlorine or chloramine process of GSH will not adversely affect the analysis of CX_3_R-DBPs, under which Cl_2_ and NH_2_Cl can be completely quenched. GSH has an obvious destructive effect on HNMs, and they should be immediately analyzed without adding a quencher, but GSH has little effect on the stability of THMs, HAAs, HALs, HKs, HANs, and HAMs. Among them, 1,1,1-TCP, TCAN, and TCNM should be analyzed as soon as possible to avoid rapid hydrolysis. A comparison of the negative effects of four quenchers on TOX determination: sodium thiosulfate > sodium sulphite > GSH > NAC. GSH is therefore an ideal quencher for THMs, HAAs, HALs, HKs, HANs, HAMs, and TOX analysis [6].

### 3.3. 1,3,5-Trimethoxybenzene (TMB)

As a new quenching agent, 1,3,5-Trimethoxybenzene (TMB) is used to preserve redox-unstable DBPs. For quenching free chlorine and free bromine, TMB has been proven to be an effective quencher. TMB does not affect the stability of eight known DBPs (TCNM, TCAL, CAN, DCAN, TCAN, BAN, DBAN, and TBAL) [30]. TMB does not degrade unstable DBP in the presence of conventional quenchers, and using TMB as a quencher provides the additional benefit of being able to quantify residual free chlorine and free bromine by separately measuring 2-Cl-1,3,5-trimethoxybenzene (Cl-TMB) and 2-Br-1,3,5-trimethoxybenzene (Br-TMB) in quench samples. However, since Cl-TMB and Br-TMB affect the TOX content of the quenched sample, TBM is not a suitable free halogen quencher in samples that are subsequently analyzed for TOX [30].

### 3.4. Comparison of Traditional and Emerging Residual Chlorine Quencher

Traditional quenching agents are mainly inorganic, but the new ones are organic. The traditional ones are cheap and easy to transport and preserve, but the new ones are relatively expensive and not easy to preserve (Table 1). For traditional quenchers, the quenching mechanism is mainly based on redox reactions, but the new reaction mechanism is more diverse. Among the traditional inorganic quenchers, sodium thiosulfate, sodium sulfite, and ammonium chloride will cause degradation of organic DBPs, and ascorbic acid will cause degradation of chlorate and MX, but the new quenchers are now more friendly to most of the organic DBPs.

## 4. Reaction of Residual Chlorine Quenchers with Chlorine

### 4.1. Traditional Residual Chlorine Quenchers

**Scheme 1:**
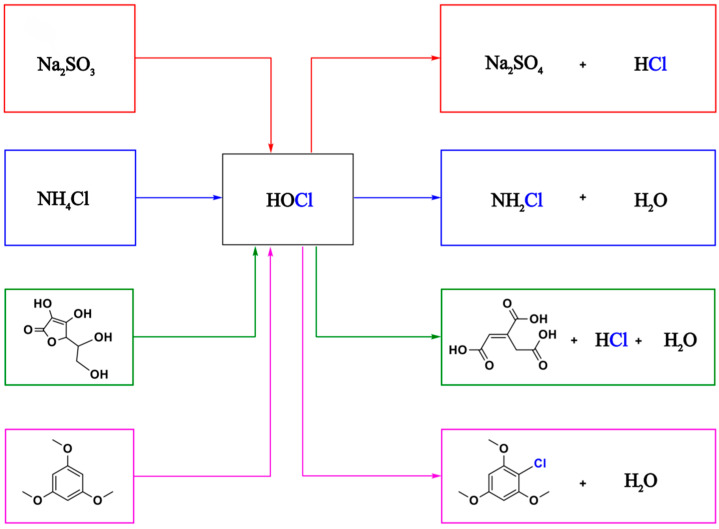
The reaction of chlorine quenchers with HOCl.

For three common chlorine quenchers, their reactions with free chlorine are shown in Scheme 1 and the following reactions [31,32,33]:

Sodium sulfite

Na_2_SO_3_ + HOCl → Na_2_SO_4_ + HCl (1)

Ammonium chloride

NH_4_^+^ + HOCl ↔ NH_2_Cl + H_2_O (2)

Ascorbic acid

C_6_H_8_O_6_ + HOCl → C_6_H_6_O_6_ + HCl + H_2_O (3)

In addition, Basu et al. (2011) showed that, for several quenchers, organic and inorganic substances may affect the speed and integrity of chlorine quenching [34]. Therefore, different quenchers should be selected to quench the residual chlorine.

The EPA Method 551.1 and Standard Methods recommend using large doses of the quenching agent at molar concentrations in the order of 10–100× the residual chlorine concentration under typical disinfection conditions [35]. However (and with the exception of ammonium chloride), lower doses are becoming more common in DBP monitoring and research, in the order of 1.2–2× the residual chlorine concentration, to minimize any potential impact of the quenching agent on DBP analysis [4,21,35,36,37].

All quenchers reduced the chlorine concentration by at least 85% within 5 s at a 1:1 ratio. However, only ascorbic acid was able to quench chlorine to below the detection limit (0.1 mg/L) at this ratio. This effectiveness from ascorbic acid was expected, as it has been previously reported to react very quickly with chlorine, quenching to below detection within <1 s in ultrapure water [32]. In comparison, sodium sulfite required between a 20 and 30% molar excess to quench chlorine to below detection (doing so within 5 s), and ammonium chloride did not quench chlorine to below detection for any of the molar ratios tested [5].

There have been numerous studies discussing the inhibitory effects of organic matter on dechlorination [38,39,40], and Basu et al. (2011) (as mentioned earlier) showed that inorganic matter may slow ascorbic acid and sodium sulfite dechlorination, depending on the quenching agent-to-chlorine ratio [34]. It may also be that dissolved oxygen oxidizes some sodium sulfite and ascorbic acid with the assistance of unknown natural catalysts in the water samples (at different rates) before they can react with chlorine, decreasing the speed at which chlorine is reduced and lowering the apparent rate coefficient between the quencher and chlorine (U.S. EPA 2000). It is not clear that this was the case in the current work, as the reaction between oxygen and sulfite is thought to proceed relatively slowly with a half-life of several hours to days [41].

The rate of oxidation of SO_3_^2−^ with HOCl is more than four orders of magnitude faster than the rate with OC1^−^. A shift in the mechanism from O atom transfer for OC1^−^ to Cl^+^ transfer for HOCl is proposed to account for the huge increase in reactivity. Below pH 11, the rate of oxidation can be limited by proton-transfer reactions. A reactive intermediate species, HOCl-SO_3_^2−^, is proposed, which further transform to ClSO^3−^. The HOCl reactivity with SO_3_^2−^ > I^−^ > Br^−^ is highly dependent on the nucleophilicity of these anions [31].

### 4.2. Emerging Residual Chlorine Quenchers

NAC can be used as a quenching agent to control DBPs content after chlorine and chloramine disinfectants. The basic reaction process is that the sulfhydryl group (R-SH) and the amino group (R-NH_2_) in NAC are the first and second reaction sites of electrophilic attack on chlorine and NH_2_Cl, respectively.

GSH is quenched by the same reaction principle as NAC. When the molar ratio of chlorine to GSH is less than 0.5, the formation of CX_3_R-DBPs can be ignored, and free chlorine and monochloramine can be completely quenched under this condition.

Both NAC and GSH belong to RSCs, and both have sulfur groups, which are the key position to react with residual disinfectants as quenchers. According to previous studies, the sulfhydryl group (RSH) and the amino group (R-NH_2_) in NAC and GSH are the first and second reaction sites for electrophilic attack by Cl_2_ or NH_2_Cl, respectively [42,43]. During the chlorination or chloramination of NAC or GSH, the sulfhydryl group is first converted to the sulfenyl chloride group (RSCl) by electrophilic substitution, and then further hydrolyzed to sulfenyl acid (RSOH) as the initial product [44]. RSOH can then be rapidly hydrolyzed and oxidized to the corresponding sulfinic acid (RSO_2_H) and sulfonic acid (RSO_3_H). Both chlorination and chloramination can achieve this reaction [42,43]. However, HClO is orders of magnitude more reactive toward amine groups than NH_2_Cl [11,29,30,31,32,33,34,35,36,37,38,39,40,41,42,43,44], and the stoichiometric ratio of NH_2_Cl to NAC or GSH is lower than HClO for these compounds. While the much higher reactivity of chlorine and chloramine towards the reduced sulfur groups in NAC and GSH protects other functional groups (e.g., alkyl, amine, and amide) that are responsible for the formation of CX_3_R-DBPs [23,24]. NAC and GSH thus maintain the stability of DBPs and serve as quenchers of choice prior to analysis.

Lau et al. (2018) suggested 1,3,5-trimethoxybenzene (TMB) as an effective quenching agent for free chlorine and free bromine, without affecting the stability of DBPs [30]. When TMB is present in sufficient excess, free chlorine can be quenched and Cl-TMB, a single stable product, can be simultaneously formed [45]. When free bromine is present, such as in chlorinated water containing bromides, TMB also reacts with free bromine to form Br-TMB, a single stable product [46]. Quantification of the single halogenated product of TMB allows researchers to selectively determine the concentration of free chlorine and free bromine in aqueous solutions, since the reaction products of TMB with free chlorine and bromine can be analyzed by gas chromatography-mass spectrometry (gc-ms). This method not only allows the removal of residual chlorine and bromine from water to avoid their further conversion into disinfection by-products, but also allows the determination of free chlorine and bromine in water.

## 5. Mechanism of the Degradation of DBPs Caused by the Quencher

**Scheme 2:**
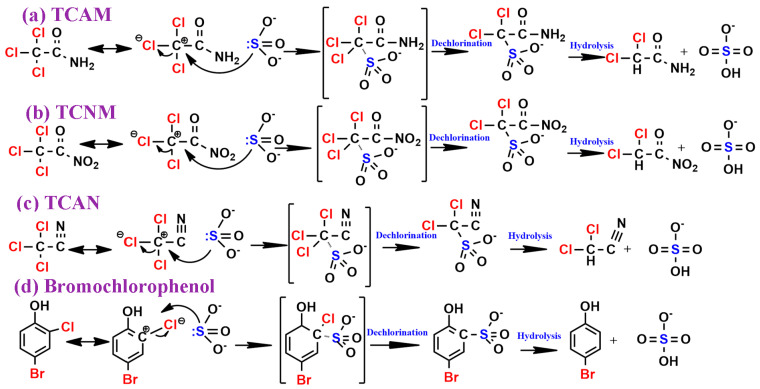
The reaction mechanism of sulfite with TCNM, TCAN, TCAM, and bromochlorophenol.

Among the studied chlorine quenchers, sulfite has a strong reducing capacity, which can cause the dehalogenation of DBPs in water. In addition, the reaction mechanisms between sulfite and phenolic DBPs, HAMs, and HNMs have been published [11,13,25]. For other traditional and emerging chlorine quenchers, although a number of studies have indicated that they caused the degradation of DBPs, the mechanism has not been provided until now [4,5]. For TCNM, TCAN, TCAM, and bromochlorophenol, the reaction mechanisms with sulfite are shown in Scheme 2. As shown in Scheme 2, the dehalogenation of TCNM, TCAN, TCAM, and bromochlorophenol was caused by nucleophilic substitution reaction. For TCNM, TCAN, TCAM, and bromochlorophenol, because of the higher electron-withdrawing capacity of chlorine atoms, carbocation is first formed, and then they will react with sulfite to form intermediate products due to sulfite having lone pair electrons. The intermediate products are not stable, due to which it is easy to form a more stable dechlorinated product. Finally, the dechlorinated products will be hydrolyzed to form sulfate, DCAN, DCNM, DCAM, or bromophenol.

## 6. Suggestions for Selecting Residual Chlorine Quenchers

An ideal chlorine quenching agent should quickly eliminate the residual chlorine and not affect the measured DBP concentration by reacting with DBP or interfering with the analytical method. The selection and use of quenching agents should meet the following four conditions: rapid and complete reaction with the residual disinfectant, chemical inertness to DBPs or other analytes of interest, negligible interfering effects in the analysis of DBPs or other analytes of interest, and an undetectable signal of the compound or its reaction products [10].

For traditional chlorine quenching agents, sodium sulfite has no significant effect on any inorganic DBPs, and is the best quencher for inorganic DBPs. Furthermore, ascorbic acid is recommended to quench most of the organic DBPs. For emerging chlorine-quenching agents, NAC, GSH, and TBM can be used as ideal chlorine quenching agents for most of the known organic DBPs. However, TBM cannot be used for TOX analysis.

## Figures and Tables

**Table 1:** Comparison of the advantages and disadvantages of traditional and emerging chlorine quenchers.

Quenching Agents		Advantages	Disadvantages	Applicable DBPs	Not Applicable DBPs
**Traditional quenching agents**	Ascorbic acid	Best agent for most of known organic DBPs; Reduce cytotoxicity Simple, cheap, and stable	Degradation of inorganic DBPs (chlorate, chlorite, and bromate) Degradation of some of organic DBPs	All of THMs, HAA, and TOX Most of HANs, HKs, HALs, HAMs, and phenolic DBPs	TCNM, TCAN, Chlorite, MX, BDCNM, DBCNM, DBAM, TBAL, inorganic DBPs
	Sodium sulfite	Best agent for inorganic DBPs, MX, THMs, and HAAs Simple, cheap, and stable	Caused degradation of most of priority DBPs and emerging DBPs	THMs, HAAs, chlorate, chlorite, bromate, inorganic DBPs, and MX	Most of HNMs, HANs, HKs, HALs, and HAMs Halopropanes, halopropylenes, halopropanes, and chloropropionitrile TOX
	Ammonium chloride	Good agent for most of organic DBPs Simple, cheap, and stable	Converts free chlorine to combined chlorine, leading to an increase in TOX Reduce MX	All of THMs, HAAs, and TOX Most of HANs, HKs, HALs, HAMs, and phenolic DBPs	TCNM, DBAM, MX
**New quenching agents**	N-Acetylcysteine	Good agent for most DBPs Simple, cheap, and stable	With a garlic-like odor; Moisture-attracting	All of THMs and HAAs Most of HANs, HALs, HKs, HNMs, HAMs, and TOX	DCNM, TCNM
	Glutathione	Stable for most DBPs	High cost Complex and time-consuming in preparation and operation	Same as n-acetylcysteine	DCNM, TCNM
	1,3,5-Trimethoxybenzene	Good for redox unstable DBPs, free chlorine, and bromine analysis	Can be disturbed by chloramines Impact TOX	TCNM, TCAL, CAN, DCAN, TCAN, BAN, DBAN, and TBAL	TOX

## Data Availability

Not applicable.

## Abbreviations

BCAL	Bromochloroacetaldehyd
BDCA	Bromodichloroacetaldehyde
BDCAL	Bromodichloroacetaldehyde
BDCNM	Bromodichloronitromethane
DBAM	Dibromoacetamide
DBAN	Dibromoacetonitrile
DBCNM	Dibromochloronitromethane
DBPs	Disinfection by-products
DCAA	Dichloroacetic acid
DCAL	Dichloroacetaldehyde
DCAN	Dichloroacetonitrile
DCNM	Dichloronitromethane
GSH	Glutathione
HAAs	Haloacetic acids
HALs	Halogenated acetaldehyde
HAMs	Haloacetamides
HANs	Haloacetonitriles
HKs	Halogenated ketones
HNMs	Halonitromethanes
MX	Halofuran
NAC	N-acetylcysteine
TBAL	Tribhloroacetaldehyde
TCAL	Tricromoacetaldehyde
TCAM	Trichloroacetamide
TCAN	Trichloroacetonitrile
TCM	Trichloromethane
TCNM	Trichloronitromethane
THMs	Trihalomethanes
TMB	1,3,5-trimethoxybenzene
TOX	Total Organic Halogen
1,1,1-TCP	1,1,1-trichloropropanon
1,3-DCP	1,3-dichloroacetone
